# Health technology assessment in the Balkans: opportunities for a balanced drug assessment system

**DOI:** 10.1080/13102818.2014.978636

**Published:** 2014-11-26

**Authors:** Dávid Dankó, Guenka Petrova

**Affiliations:** ^a^ Institute of Management, Corvinus University of Budapest, Budapest, Hungary; ^b^ Department of Social Pharmacy, Faculty of Pharmacy, Medical University of Sofia, Sofia, Bulgaria

**Keywords:** reimbursement of medicines, pricing of medicines, HTA, Balkan region

## Abstract

Countries in the Balkan region use pharmaco-economic data for decisions about the inclusion of new pharmaceuticals into their positive drug lists, but no predefined frameworks are used and resources for health technology assessment (HTA) are limited. The goal of this analysis is to investigate into possible development directions for the HTA system in the region, and provide some practical recommendations for a sustainable model. For this purpose, the main factors currently influencing HTA in Balkan countries are briefly presented, and possible development strategies are compared. A resource-saving balanced assessment approach is proposed. It is aligned with available resources and capabilities, and helps access to new pharmaceuticals while ensuring the transparency of decision-making processes and the stability of the pharmaceutical budget.

## Introduction

Although not a precisely defined and universally accepted geopolitical term, the ‘Balkans’ is commonly used to refer to countries in southern Central Europe which share several historical, cultural, political and economic characteristics. Bulgaria, Croatia, Greece, Romania and Slovenia are member states of the European Union (EU), while Albania, Bosnia and Herzegovina, Macedonia, Montenegro (Crna Gora) and Serbia do not have membership yet. Most Balkan states are middle-income countries which, at the same time, show large differences in economic development. Common characteristics are, however, small national economies, which are highly exposed to economic cycles in the Eurozone, dependence on imports of highly processed products, as well as unemployment and the resulting large numbers of emigrant workforce. Purchasing power adjusted per capita gross domestic product (GDP) values range from 28,600 USD (Slovenia) to 18,100 USD (Croatia) and 8000 USD (Albania). Many economies of the region have recently shrunk as a consequence of the economic crisis.[1]

Health systems in most Balkan countries are characterized by mandatory national health insurance. Most health care institutions are publicly owned. The health insurance system is managed by national health insurance funds. Public health care expenditures amount to 2.6%–6.9% of total GDP (Bosnia and Herzegovina, Croatia, Greece, Montenegro, Serbia and Slovenia have higher values; Albania, Bulgaria, Macedonia and Romania have lower values), whereas public pharmaceutical expenditures (PPE) reach only 0.6%–0.9% of the total GDP.[2] Within the EU, this puts Bulgaria and Romania at the bottom of the scale in terms of public pharmaceutical spending, together with Poland and Hungary. Factors underlying low PPE are limited financial resources, relatively high co-payments, widespread use of over-the-counter medications, as well as the delayed arrival of innovative pharmaceutical products to the countries.

In an average Balkan country, pharmaceuticals are reimbursed either from the outpatient pharma budget (prescription budget), or from the hospital budget. Central tenders apply in some markets for more expensive pharmaceuticals. In order to be listed in reimbursement formularies, pharmaceuticals normally need their prices to be registered or authorized and their reimbursement rate to be defined, typically by the Ministry of Health. Decision-makers currently require only basic pharmaco-economic (PE) data for pricing and listing decisions. Drug assessment generally covers the evaluation of efficacy and therapeutic effectiveness, safety as well as PE considerations. As of 2013, few explicit decision guidelines (e.g. health technology assessment (HTA) guidelines, threshold values, scoring systems) are used.[3]

The goal of this work is to present an analysis of the commonalities and differences influencing the pricing and reimbursement of medicines in the Balkan countries and to propose a theoretical balanced model for organizing both processes.

## Materials and methods

This study is based on a theoretical, observational process analysis of the established HTA models in the world and the pricing and reimbursement practice in the field of medicines in the Balkan countries. On the basis of this analysis, a balanced model for the assessment of innovative medicines is proposed that could be customized in line with local country specificities. The underlying conceptual framework of balanced assessment for middle-income countries is previously discussed in [4].

## Results and discussion

### Current context and shortcomings of HTA

The current shortcomings of HTA in the Balkans are typical for middle-income countries [4] and are mostly related to the lack of use of clear HTA methodologies and rules, the quality of local epidemiology and health statistics data.

#### Methodologies

During the pricing and listing process, few explicit HTA frameworks and guidelines are used. Decision-making is based on expert opinion and regulatory requirements. In most countries, key requirements have not been set for cost-effectiveness analysis and, in some cases, for budget impact calculations, yet. Consequently, there are large variations in the PE data submitted by manufacturers, and the robustness of the subsequent assessment is dependent upon the quality of the dossier.

#### Local data availability

Local epidemiology data are either missing or incomprehensive, or they may be inconsistent or significantly distorted by biases. Epidemiology data are not collected for certain therapeutic areas. In addition, patient pathways are often undefined, making it very complicated to assess the performance of health care subsystems. Limited availability of reliable local data and undefined patient pathways also result in international PE models prepared for new medicines being very difficult to adapt.

#### Experts and capabilities

The number of trained HTA experts is relatively limited in some of the larger countries of the region (Greece, Serbia, Croatia, Bulgaria) and very low in other countries (Romania, Albania, Bosnia and Herzegovina, Macedonia),[5] although basic PEs education is available for pharmacy students at the main medical universities in the region, particularly Zagreb, Belgrade, Sofia, Varna and Bucharest. As a result, there is a small number of HTA experts working as consultants for the authorities and with pharmaceutical companies.

### Possible HTA models for the Balkan region (conceptual background)

In one possible definition,[6] HTA is the ‘systematic evaluation of medical technologies regarding their effectiveness, appropriateness, efficiency as well as social and ethical aspects and implications’. In another definition,[7] ‘healthcare technology is defined as prevention and rehabilitation, vaccines, pharmaceuticals and devices, medical and surgical procedures, and the systems within which health is protected and maintained. Technology assessment in health care is a multidisciplinary field of policy analysis. It studies the medical, social, ethical and economic implications of development, diffusion and use of health technology’. Thus, HTA has a decision-support role helping in reimbursement policy-making and in taking higher level political decisions in health care by providing timely, accurate and sound information on medical technologies for decision-makers. HTA is both linked to pricing and listing decisions and subsequent reimbursement reviews.

As mentioned above, HTA generally covers pharmaceuticals, medical devices as well as all clinical procedures, including surgical interventions and diagnostics. In most countries, HTA focuses on pharmaceuticals and medical devices, because these are the most standardized – and ‘calculable’ – technologies, they pose a high burden on social insurance systems, and their social impact and visibility are high.

In the pharmaceutical domain, the key question to which HTA should provide an answer is

“Is it worth spending public money on a medicine?

If yes, how much, and for which patients?”

**Figure 1.**
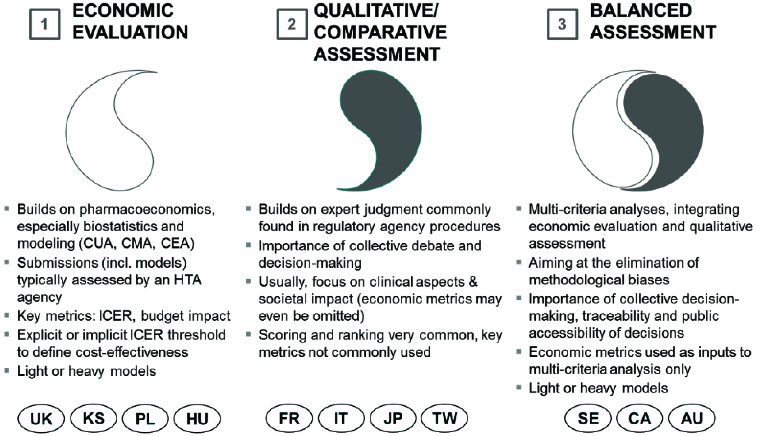
Three paradigms (archetypes) of health technology assessment.[4,8] Country abbreviations are according to ISO 3166-1 standard. Note: Countries are indicated at the paradigm they are closest to, but cannot be regarded as archetypes themselves as they show very important individual variations even within a paradigm.

Over the years, three main HTA paradigms (archetypes) have evolved across major health care markets [8] (Figure 1, described in detail in,[4]) and it has also become apparent that paradigms are not equally applicable in counties with different levels of development.

#### Economic evaluation

In this paradigm, the assessment of pharmaceuticals is quantitative and heavily reliant on statistical methods. It is commonly focused on cost-effectiveness and budget impact. For the Balkan states, which can be classified as middle-income countries, economic evaluation can actually prove to be a significant barrier in patient access to new medicines for the reasons highlighted in [4,9–11].

#### Qualitative assessment

The nature of this paradigm is more similar to the regulatory assessment approach prior to marketing authorization. Assessment of pharmaceuticals is focused around therapeutic benefit but other criteria may also be used, e.g. cost-effectiveness itself, side-effect profiles or convenience of use. Sometimes assessment is complemented by a scoring system and a categorization of pharmaceuticals. In a setting of severe budget constraints, such as that found in middle-income Balkan countries, qualitative assessment is considered insufficient because it tends to underplay financial aspects.[4]

#### Balanced assessment

Balanced assessment is a multi-criteria approach, which aims at integrating the strengths of economic evaluation and qualitative assessment while eliminating their conceptual and methodological shortcomings. In general, balanced assessment can take into account cost-effectiveness, budget impact, therapeutic value added and alignment with local health policy (and social) priorities.

### Balanced assessment system in the Balkans: framework and process

**Figure 2.**
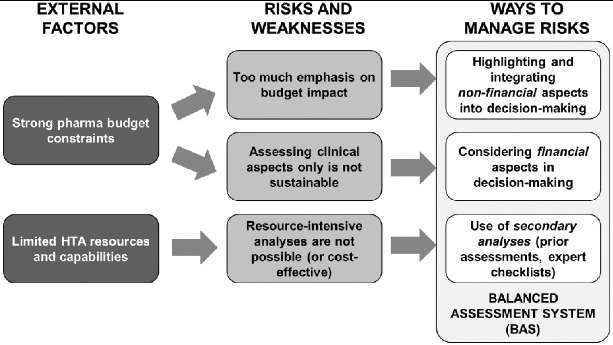
Reasoning behind a balanced assessment system (BAS) in middle-income countries.[4]

**Figure 3.**
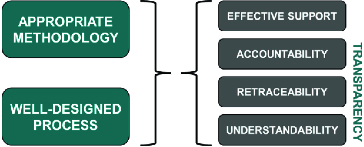
Appropriate methodology and a properly designed process together lead to effective support and transparency.[4]

Based on the characteristics described in [4], it may be appropriate in the Balkan states to opt for a balanced assessment system (BAS), which should be a sustainable and implementable compromise combining financial and non-financial aspects in a way aligned with local resources and capabilities (Figure 2). A BAS for reimbursement decision-making requires an appropriate combination of methodology and evaluation process (Figure 3). The methodology should be mostly based on secondary analyses and should also be easy to use both by persons responsible for evaluation and by decision-makers. The assessment process must be transparent, i.e. traceable decisions, understandable considerations and participants held accountable for their decisions (for more details see [4]).

**Figure 4.**
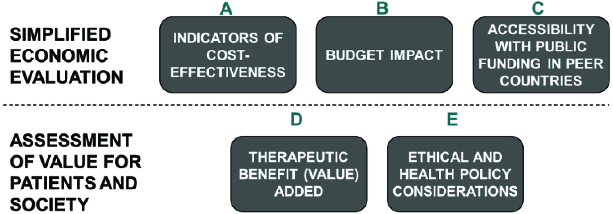
Balanced assessment system (BAS) in middle-income countries.[4]

Regarding the methodology, Balkan states may consider a balanced evaluation grid based on multiple criteria, which is aligned with the specific characteristics of middle-income countries. An example of the evaluation grid is shown in Table 1. The grid subsumes five critically relevant aspects of a pricing and listing decision under two categories (Figure 4): simplified economic evaluation (indicators of cost-effectiveness, budget impact, accessibility with public funding in peer countries) and assessment of value for patients and society (therapeutic value/benefit added, ethical and health policy considerations).

**Table 1.** Example of a BAS evaluation grid for a hypothetical country in the Balkan region (developed on the basis of [4]).

Simplified economic evaluation			
(1) Indicators of cost-effectiveness	1.1. The medicinal product has been found to be cost-effective by one or more leading international assessment bodies in the indication submitted for reimbursement, or the medicinal product has been found to be cost-effective by a local study for the national health care system in the indication submitted for reimbursement.	High weight	Mutually exclusive with 1.2.
	1.2. The medicinal product has been found to be cost-effective by one or more leading international assessment bodies in part of the indication submitted for reimbursement, or the medicinal product has been found to be cost-effective by a local study for the national health care system in part of the indication submitted for reimbursement.	Medium weight	Mutually exclusive with 1.1 and has lower value.
(2) Accessibility with public funding in peer countries	2.1. The medicinal product is reimbursed through public funds in at least five European countries.	Medium weight	Mutually exclusive with 2.2.
	2.2. The medicinal product is reimbursed through public funds in less than five European countries.	Low weight	Mutually exclusive with 2.1 and has lower value.
(3) Budget impact	3.1. Local budget impact analysis has found the medicine to reduce direct health care expenditures (considering all relevant budgets).	High weight	Mutually exclusive with 3.2.
	3.2. Local budget impact analysis has found the medicine to reduce indirect health care expenditures.	Medium weight	Mutually exclusive with 3.1 and has lower value.
Assessment of the value for patients and society			
(4) Therapeutic value added	4.1. The medicinal product has been found to offer significant therapeutic benefit by one or more leading international assessment bodies, or the medicinal product offers significant improvement over the comparator therapy/ies in the primary endpoint as evidenced by at least one phase III randomized clinical trial.	High weight	Mutually exclusive with 4.2.
	4.2. The medicinal product has been found to offer modest or medium therapeutic benefit by one or more leading international assessment bodies, or the medicinal product offers modest or medium improvement over the comparator therapy/ies in the primary endpoint, or substantial improvement in a secondary endpoint, as evidenced by at least one phase III randomized clinical trial.	Medium weight	Mutually exclusive with 4.1 and has lower value.
	4.3. The medicinal product has a significantly more favourable side-effect profile than the comparator therapy/ies, considering frequency, severity and health burden of side effects.	Medium weight	Mutually exclusive with 4.3.
	4.4. The medicinal product has a somewhat more favourable side-effect profile that the comparator therapy/ies, considering frequency, severity and health burden of side effects.	Low weight	Mutually exclusive with 4.4 and has lower value
	4.5. The manufacturer has been able to substantiate superior real-life therapeutic effectiveness for the medicinal product in comparison to the real-life effectiveness of comparator therapy/ies, in international studies or data analyses covering sufficient patient numbers.	Low weight	
	4.6. The manufacturer has been able to substantiate that the medicinal product improves ease-of-use (convenience) for patients in comparison to comparator therapy/ies.	Low weight	
	4.7. The active substance has been in established use internationally for at least 15 years in the same pharmaceutical form and dose strength.	Medium weight	
			
(5) Ethical considerations and health policy priorities	5.1. The reimbursement application is submitted in an indication which has previously been declared a primary public health priority by state authorities.	Low weight	
	5.2. The medicinal product has been registered for the treatment of an orphan disease or paediatric indication.	Low weight	
	5.3. No new active substance has been admitted into reimbursement in the relevant therapy area for the last 24 months.	Medium weight

The following specifics are relevant for better understanding of this evaluation grid:

Cost-effectiveness is acknowledged either directly or indirectly (i.e. based on prior assessments by international HTA agencies). Whenever prior assessments can be found in comparable markets, these should be considered. Besides European recommendations, guidance from non-European countries can also be taken into account, resources permitting. Based on the considerations above, pricing and reimbursement bodies should not link reimbursability to the submission of health economics models but have the right to assess models if these are available.

As regards accessibility with public funding in peer countries, it is better to avoid randomly, or arbitrarily, selected baskets of reference countries. Instead, in the Balkan region, which is part of Europe, the number of EU member states where the assessed medicine is already available with public funding could be taken into consideration. This would help overcome biases resulting from any arbitrary basket of reference countries.

Budget impact should be evaluated across all sub-budgets of national health insurance funds (including pharma budget(s), hospitals, primary care, sick leaves etc.).[4] This can be done either parallel to the assessment of cost effectiveness and accessibility with public funding or, alternatively, at a later stage of the decision-making process (see below). In the latter case, the budget impact assessment can be carried out in conjunction with negotiations between the decision-makers and the manufacturer about cost/risk-sharing modalities.

The components of therapeutic value/benefit added, in a BAS, should be explicitly analysed and assessed (rather than being condensed into a high-level indicator such as quality-adjusted life-years [QALY]). At least the following components of therapeutic value/benefit added should be recognized: superior clinical outcome (higher efficacy), side-effect profile, ease-of-use (convenience) and, eventually, evidence on real-life therapeutic effectiveness. These components are likely to be included with different weights.

Ethical and health policy considerations must be covered via a set of country-specific, relevant criteria.

The design of the example evaluation grid in Table 1 is based on the assumption that public payers generally tend to prefer two categories of medicines: (1) those with considerable therapeutic value added, which are normally sold at a premium price; and (2) such that are non-inferior to an already reimbursed comparator but save resources either in the pharma budget itself, or more generally in health insurance funds.[4] Table 1 is an exemplary grid which can serve as the basis for balanced evaluation in the Balkan countries; there are several possible modifications. In the grid, each of the aspects (criteria) above is represented through statements and each statement is complemented by an individual score allocated to it. If the medicinal product meets the criteria formulated in the aspect statement, it will receive the individual score linked to that aspect; otherwise it will receive an individual score of zero for that aspect. Individual scores are then aggregated into a total score and the listing decision will strongly correlate with the total score. Possible decision rules are shown in Table 2: in this example, there is differentiation between unconditional reimbursement, conditional reimbursement and no reimbursement.

**Table 2.** Example for decision-rules in a BAS evaluation.[4]

Total score (calculated as the simple sum of individual scores)	Decision rule
Score < first cut-off point	Not reimbursable
First cut-off point < score < second cut-off point	Conditional reimbursement with programmed reimbursement review within 18–24 months or
	reimbursement with outcome guarantee by the manufacturer
Score > second cut-off point	Unconditional reimbursement

The example grid does not contain numerical scores – these have to be agreed on in each market. It is crucial, however, that relative importance of aspect statements should keep the recommendations presented in Table 1. It is also essential that the final version of the evaluation grid should be based on the broadest available consensus. While applying, the grid must be itself ‘cost-effective’ (i.e. usable with low resources), any simplifications and generalizations that may lead to inappropriate pricing and reimbursement decisions should be avoided. Therefore, the final version of the grid should be elaborated through a series of expert discussions among ministries, health insurance funds, medical and pharmacy unions, trade associations and patient associations.

**Figure 5.**
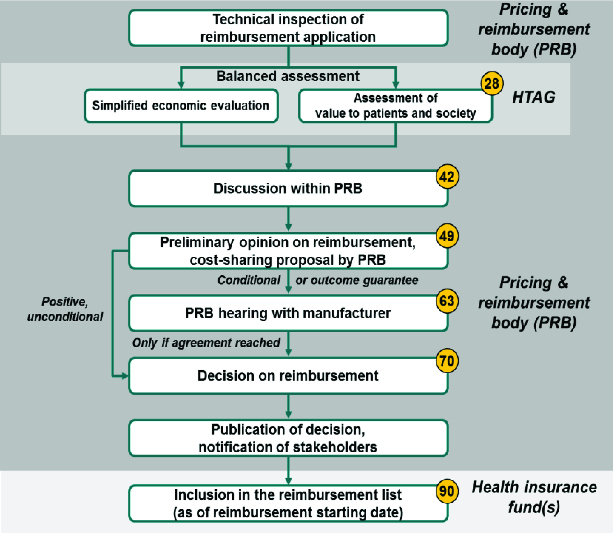
Possible simplified scheme for the evaluation and listing process in a Balkan country (modified from [4]). Notes: (1) The process only applies to pharmaceutical reimbursement decisions. Price-only applications may be managed in their current form. In the long run, administrative price setting for non-reimbursed drugs may even be abolished. (2) Numbers in circles show deadlines in calendar days. (3) Abbreviations: PRB – pricing and reimbursement body, HTAG – health technology assessment group (associated with PRB).

Regarding the evaluation and listing process, we recommend the framework illustrated in Figure 5, as discussed in detail in [4]. The deadlines along the process (in calendar days) are proposed as manageable timeframes for authorities, which do not compromise on the quality of assessment and decision-making. We point out that there may be more than one hearing (consultation) with the manufacturer but a decision must be taken after no more than three hearings. If the budget impact has not been assessed in the process yet (as part of simplified economic evaluation), it must be assessed at the stage of public hearings in a way that confidential price information remains respected and protected.

### Practical recommendations for pricing and reimbursement bodies in the Balkan region

Within the framework of a BAS, countries in the Balkan region will have latitude for customized system developments. At the same time, there are some critical factors for success

Stepwise system implementation – BAS can be applied not only for pharmaceuticals but for several other health technologies, including medical devices and diagnostics. However, excessive broadening of the scope would entail the possibility of losing focus, which is a huge risk to the social and political acceptance of the system. Therefore, a stepwise approach is strongly recommended. First, only innovative medicines applying for public funding should be subject to BAS. In a couple of years, when sufficient experience and know-how has already been accumulated, BAS can be extended to cover reimbursed non-customized (off-the-shelf) medical devices. This will require the adaptation of the evaluation grid whereby different versions will be necessary for disposable devices, devices for short-term use and devices for long-term use. In a third step, other standardized interventions can be covered. It is generally not recommended to incorporate non-standardized technologies or technologies without clear ownership.

Management of generic products – drug assessment is generally not required for bioequivalent generic products provided that a reimbursement threshold (generic price ceiling) is used, which is the case in some Balkan countries (e.g. Romania, Croatia). For non-bioequivalent generics and/or generic and biosimilar products which do not comply with the price ceiling, BAS may be a prerequisite for reimbursement.

Reimbursement reviews – BAS may not be applicable for reimbursement reviews in the same form as for new applications, as the relevant criteria may be very different. For reimbursement reviews, different aspects may be developed, taking into account that there is already an on-treatment population and thus, any de-listing or reimbursement restriction will have an impact on already initiated therapies. Furthermore, it needs to be understood that initially there may not be enough resources and capabilities to cover reimbursement reviews.

Skill development – it may help the acceptance and uptake of the new system if skill development trainings are delivered for stakeholders participating in the decision-making process. Trainings should be differentiated based on stakeholders’ role in decision-making.

Public communication – BAS can be expected to impact positively on patients, physicians and the broader society as it can facilitate access to value-added therapies while ensuring elements of financial control. In order to make this clear to the public, the purpose and the logic of the new system should be communicated to physicians, pharmacists as well as the general public.

## Conclusions

Countries in the Balkan region currently use PE evidence for pharmaceutical reimbursement decisions mostly in an unstructured way but there are clear trends to implement more formal HTA systems.

In view of the limited pharmaceutical budget and the partial lack of HTA capabilities, newly implemented formal HTA systems should ensure higher process transparency as well as access to value-added and/or cost-saving new medicines in a way that no resource-intensive assessments are necessary. For this purpose, a BAS may be a viable compromise between decision-makers’ needs and methodological reliability.

BAS models should follow a pragmatic model whereby secondary assessments are used whenever this is available, and drug assessors reach back to benchmarkable prior international evaluations as well as carefully designed expert checklists to reach decisions. The BAS system, which covers cost-effectiveness, accessibility with public funding in peer countries, budget impact, therapeutic value/benefit added as well as ethical/health policy priorities, should be embedded in a transparent and streamlined pricing and listing process. The key roles in this process will be fulfilled by pricing and reimbursement bodies and their assessment satellites, which are health technology assessment groups (HTAG's).

Initially, HTA systems in the Balkan region should be focused on new innovative pharmaceuticals seeking reimbursement. In the later stages, systems can be extended to cover certain medical devices and other standardized technologies. We envisage that a BAS implemented in such a stepwise approach can significantly contribute to the efficiency and equity of reimbursement decision-making in the region. Also, the introduction of such a system can improve transparency and lead to a sustainable health care budgets and access to medicines.
